# Revision of the family Carabodidae (Acari, Oribatida) XII. *Yoshiobodes camerunensis* sp. n. and *Rugocepheus costaricensis* sp. n.

**DOI:** 10.3897/zookeys.706.14807

**Published:** 2017-10-04

**Authors:** Nestor Fernandez, Pieter Theron, Sergio Leiva, Louwrens Tiedt

**Affiliations:** 1 National Council of Scientific and Technological Research, Argentina (CONICET). Subtropical Biological Institute (IBS). Evolutive Genetic Laboratory FCEQyN, Misiones National University. Felix de Azara 1552, 6º, (3300) Posadas Misiones, Argentina; 2 Research Unit for Environmental Sciences and Management, North-West University, Potchefstroom Campus, 2520, South Africa; 3 Fellowship, National Institute Agricultural Technology (INTA). Experimental Rural Agency, Aimogasta. La Rioja, Argentina; 4 Laboratory for Electron Microscopy, North-West University, Potchefstroom Campus, 2520 South Africa

**Keywords:** Carabodidae, *Rugocepheus*, *Yoshiobodes*

## Abstract

*Yoshiobodes camerunensis* **sp. n.**, collected in Cameroon, is the first species of this genus reported from the Afrotropical region. Diagnostic characters include lamellae terminating in a bridge and not in lamellar tips; cup-shaped bothridia, bothridial ring present; rostral setae cochleariform, smooth; lamellar setae slightly lanceolate, barbate; fifteen pairs of notogastral setae; *c_3_* lanceolate, rounded end, with longitudinal shallow grooves; other notogastral setae curved lanceolate-cochleariform. *Rugocepheus costaricensis* **sp. n.** is the third species of the genus to be described, and the first collected outside the African region. Prodorsum presents a Y-shaped structure; elevated interlamellar process, superior flat zone; lamellae lacking lamellar tips; fourteen pairs of notogastral setae; four notogastral furrows, and an unpaired elevated central area devoid of setae. Both species are described and illustrated based on adult specimens, studied by means of optical and SEM microscopy.

## Introduction

Extensive collection materials of the family Carabodidae sampled in Africa (Cameroon, Kenya, Zimbabwe, Rwanda, South Africa, Madagascar, Gabon, Comoros, Republic of the Congo, Democratic Republic of the Congo, Nigeria, Ghana); South and Central America (Argentina, Chile, Brazil, Paraguay, Uruguay, Bolivia, Peru, Ecuador, Costa Rica, Martinique, Honduras, Guadeloupe, Trinidad-Tobago), and Asia (Vietnam, China, Cambodia, Sri Lanka) are housed in the Museum national d’Histoire naturelles, Paris (**MNHN**), the Museum d’Histoire naturelles Geneva (**MHNG**) and in the senior author’s personal collection. Studies of this material have been ongoing, in parallel to the redescriptions of type material of the various genera started in 2013.

The taxonomy of the genus *Yoshiobodes* is complex. This genus comprises 12 species and is divided into three subgenera: *Yoshiobodes*, which includes eight species with Pantropical (excluding Ethiopic) and Subtropical (Holarctic Southern) distribution; *Berndobodes* with two species from Borneo, and *Dongnaibodes* with two species from Vietnam (Subias 2017). According to Reeves (1997), the type species is *Yoshiobodes irmayi* (Balogh & Mahunka, 1969), with Neotropical distribution, and the comparison of *Y. irmayi* collected from North America and from St. Lucia, West Indies, revealed that they are conspecific.

This genus is very difficult to study using optical microscopy due to their small size, cuticular microsculpture, cerotegumental layer, particular topography, and setal particularities. The complimentary use of Scanning Electron Microscopy (SEM) is fundamental to understanding and clarifying several aspects of this fascinating group of Carabodidae. The contribution by Reeves (1997) is remarkable, and the redescription of the type species *Y. irmayi* is given here for the first time including both adults and immatures, as well as SEM micrographs. *Yoshiobodes camerunensis* sp. n. is the first species of this genus found in the Afrotropical region.

The second species, *Rugocepheus costaricensis* sp. n. is described from Costa Rica. Two species of this genus are known previously from Africa, namely *Rugocepheus formosus* Mahunka, 2009 and *Rugocepheus joffrevillei* Fernandez, Theron & Rollard 2013, both from Madagascar.

## Materials and methods

The techniques used in the light and scanning electron microscopic investigations of the examined specimens follow those proposed by Fernandez et al. (2013).

The SEM observations were made using Scanning Electron Microscope FEI-Quanta Feg 250, with 10 Kv and working distant (WD) variable.

Measurements taken: total length (from tip of rostrum to posterior edge of notogaster); width (widest part of notogaster) in micrometers (μm). Leg setation studies making use of standard, polarized and phase contrast microscopes are provisional, due to the fact that only adult specimens were available for study. Setal formulae of the legs include the number of solenidia (in parentheses); tarsal setal formulae include the famulus (ε).

### Morphological terminology

Morphological terms and abbreviations used are those developed by F. Grandjean (1928–1974) (cf. Travé and Vachon 1975; Norton and Behan-Pelletier 2009 (in Krantz and Walter 2009); Fernandez et al. 2013). For the setal types those of Evans (1992); ornamentation of cuticular surfaces Murley (1951) (in Evans 1992 *op. cit.*: 9) were used.

## New taxa descriptions

### Yoshiobodes camerunensis sp. n.

Taxon classificationAnimaliaSarcoptiformesCarabodidae

http://zoobank.org/55FC7386-4819-4BF0-9F21-32AFABB393ED

Figures 1–2 , 3–5 , 6–13 , 14–20 , 21–27 , 28–35 , Table 1

#### Etymology.

The specific epithet is derived from Cameroon, country of origin of the type material.

#### Material examined.

**Holotype**. Adult female “CAM 73/3. Mt.Kala (près de Yaoundé). 800–850 m, terreau troncs pourris et litière. IV–V. 1973. Leg. G. TERRON”. Material deposited in the collection of MNHG, Switzerland, preserved in 70% ethanol. **Paratypes**. 2 adult females “CAM 73/3. Mt. Kala (près de Yaoundé). 800–850 m, terreau troncs pourris et litière. IV–V. 1973. Leg. G. TERRON”. Material deposited in the collection of MNHG, Switzerland, preserved in 70% ethanol.

#### Diagnosis.

*Setation.* Rostral setae cochleariform, smooth; lamellar setae slightly lanceolate, barbate, covered by cerotegumental layer; notogastral *c_1_*, *c_2_*, *da*, *dm*, *dp*, *lm*, *lp* curved lanceolate-cochleriform; *c_3_* lanceolate, rounded end with longitudinal shallow grooves; subcapitular *h*, epimeral, genital, anal, adanal setae spiniform. *Prodorsum*. Shallow lamellar furrow present; lamellae terminate in bridge not lamellar tips. Bothridia cup-shaped, with bothridial ring. Barbed fan-shaped sensillus. *Notogaster*: fifteen pairs of setae. Genital opening on elevated zone; deep anterior furrow in front of genital opening.

**Figures 1–2.**
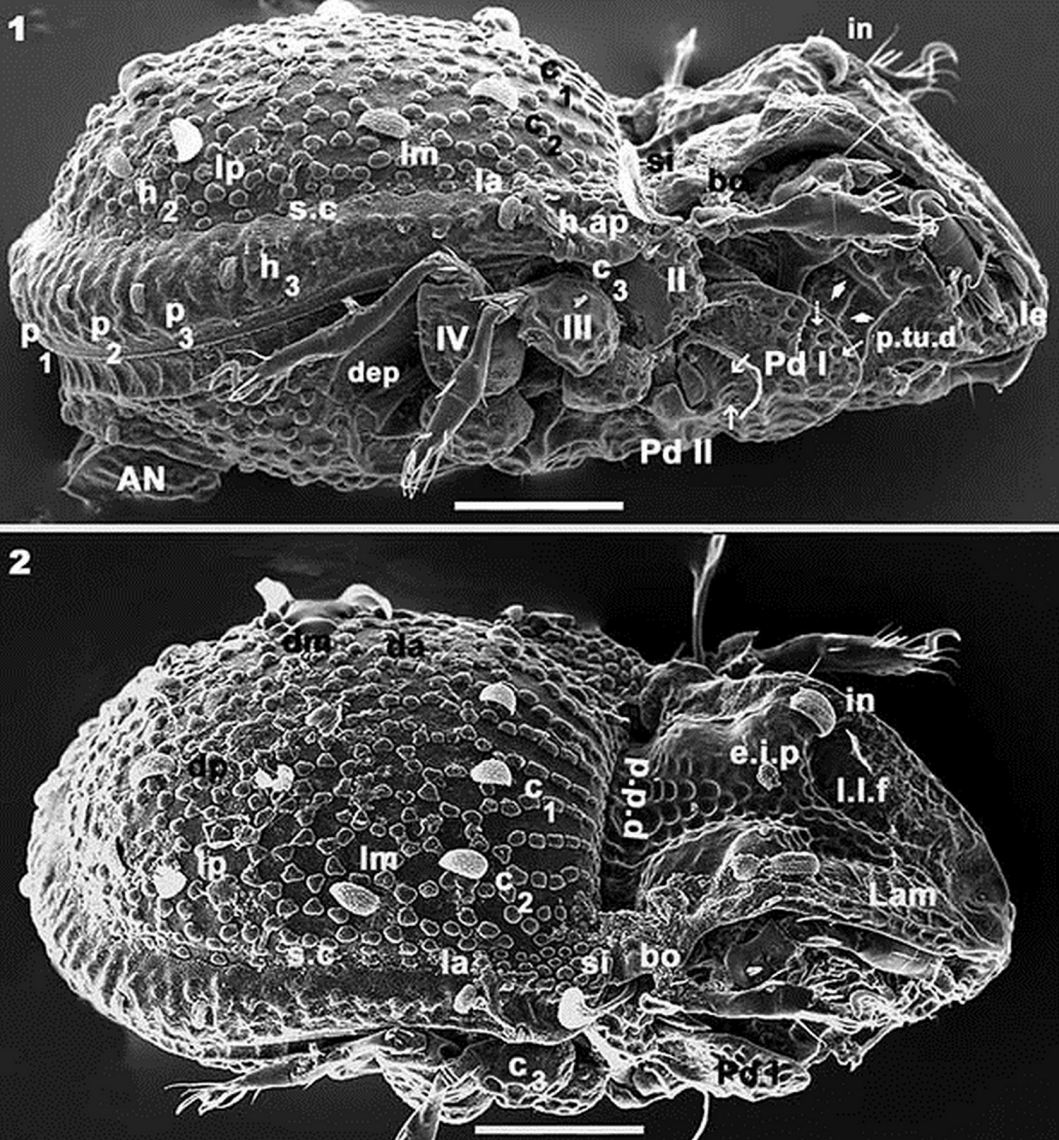
*Yoshiobodes camerunensis* sp. n. Adult, with cerotegumental layer. SEM micrographs. **1** lateral view **2** dorsal with slight lateral tilt. Scale bars: 50 μm (**1, 2**).

**Figures 3–5.**
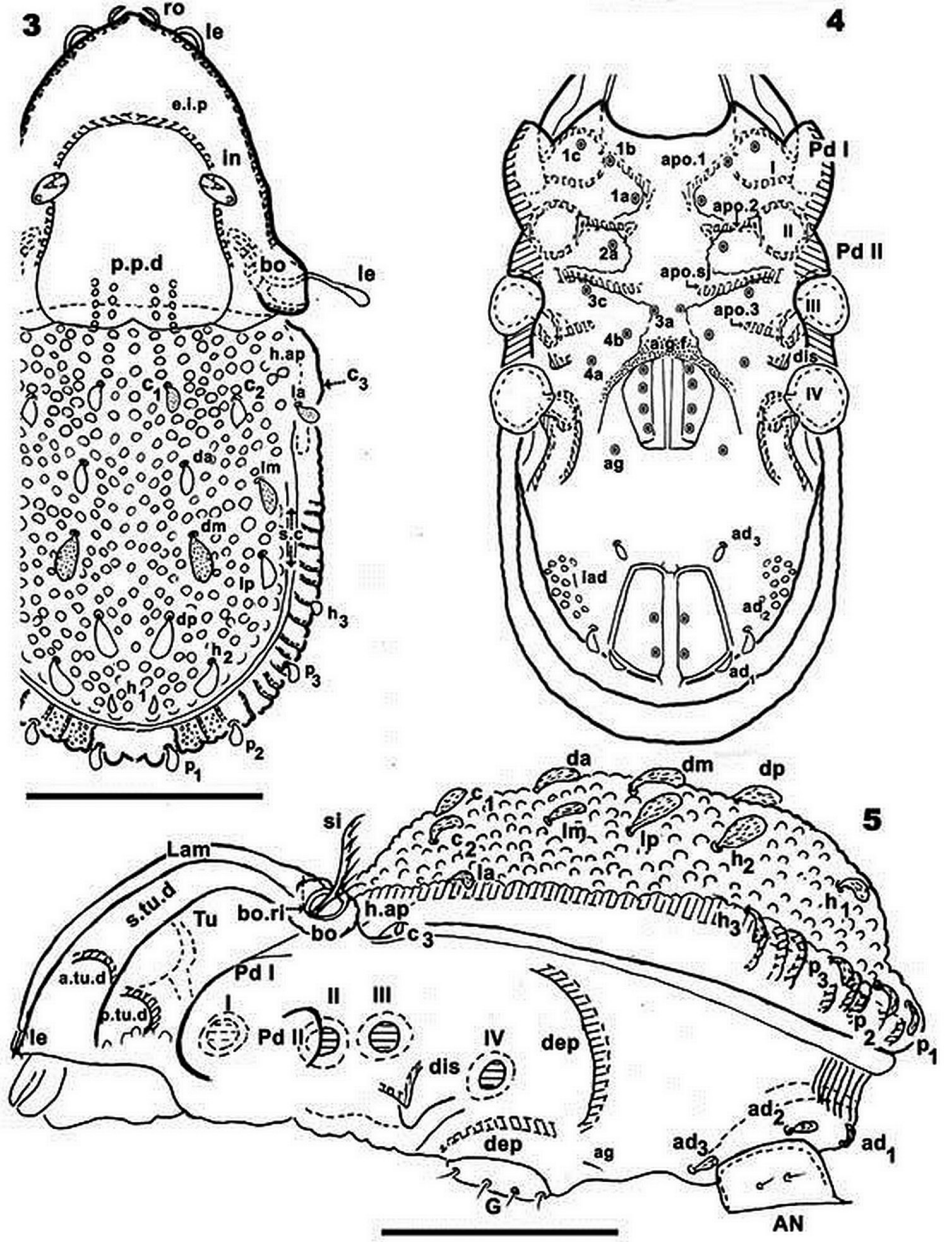
*Yoshiobodes camerunensis* sp. n. Adult, Optical Microscopy. **3** dorsal view **4** ventral view **5** lateral view. Scale bars: 45 μm (**3, 4**); 80 μm (**5**).

#### Description.

*Measurements*. SEM: length: 301 μm (296–312). Width: 130 μm (127–152) (three specimens). Light microscopy: 311 μm (301–323) × 142 μm (138–148) (two specimens) All specimens female.

*Colour*: Specimens without cerotegument, light brown, slightly shiny when observed in reflected light.

*Cerotegument*: Entire body, femora and genua of legs covered by thin layer of between 0.1–0.5 μm presenting as a polygonal network (Figures 7, 17, 19, 26, 30, 31 indicated by arrow).

**Figures 6–13.**
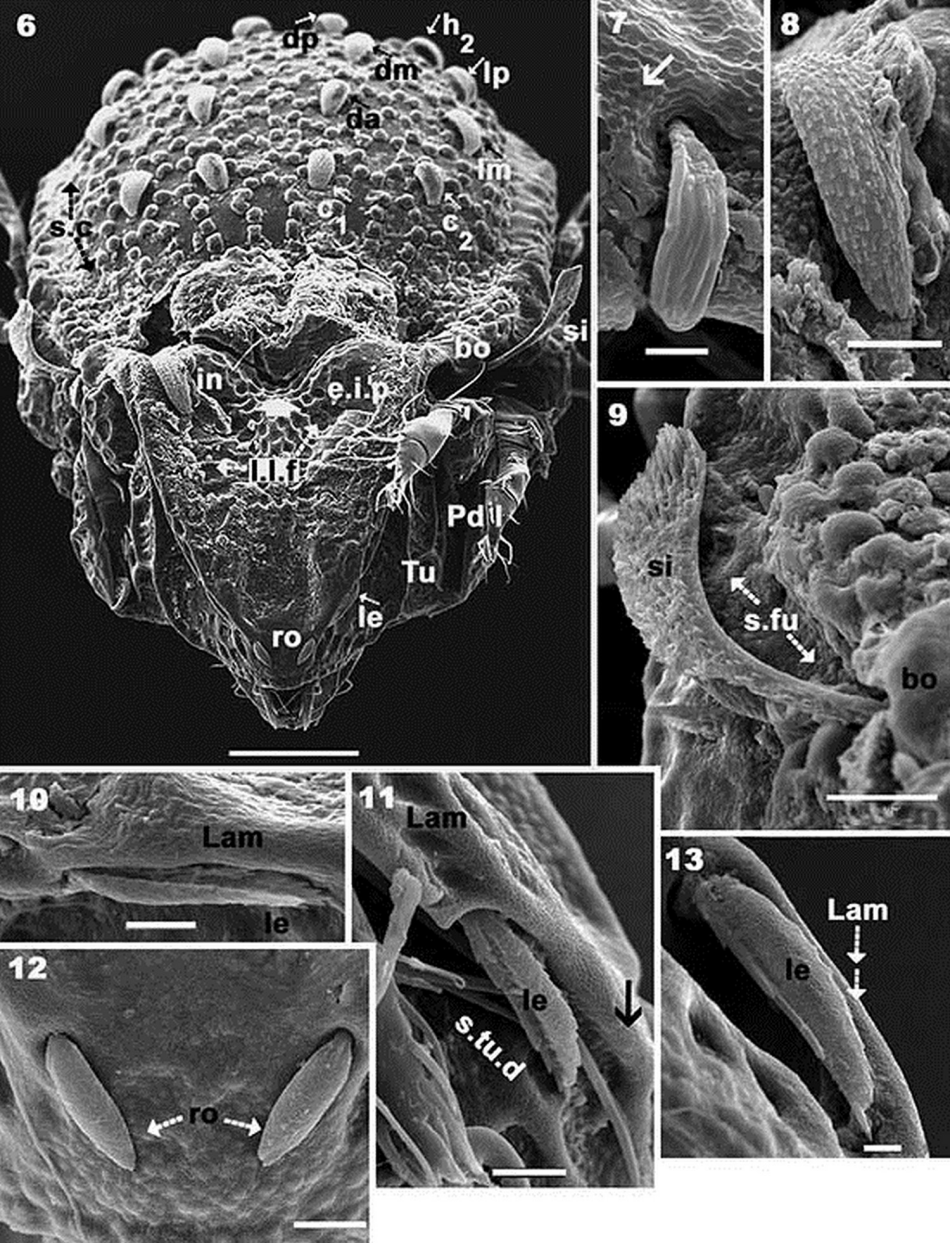
*Yoshiobodes camerunensis* sp. n. Adult with cerotegumental layer, SEM. **6** frontal view **7** *c_3_* setae **8** *in* setae **9** *si* and sensillar furrow (*s.fu*) **10** *lam* lateral with *le* setae **11** *lam*, lateral with *le* setae **12** rostral setae **13** *lam* dorsolateral with *le* setae. Scale bars: 50 μm (**6**); 2 μm (**7**); 10 μm (**8**); 10 μm (**9**); 5 μm (**10**); 10 μm (**11**); 5 μm (**12**); 2 μm (**13**).

**Figures 14–20.**
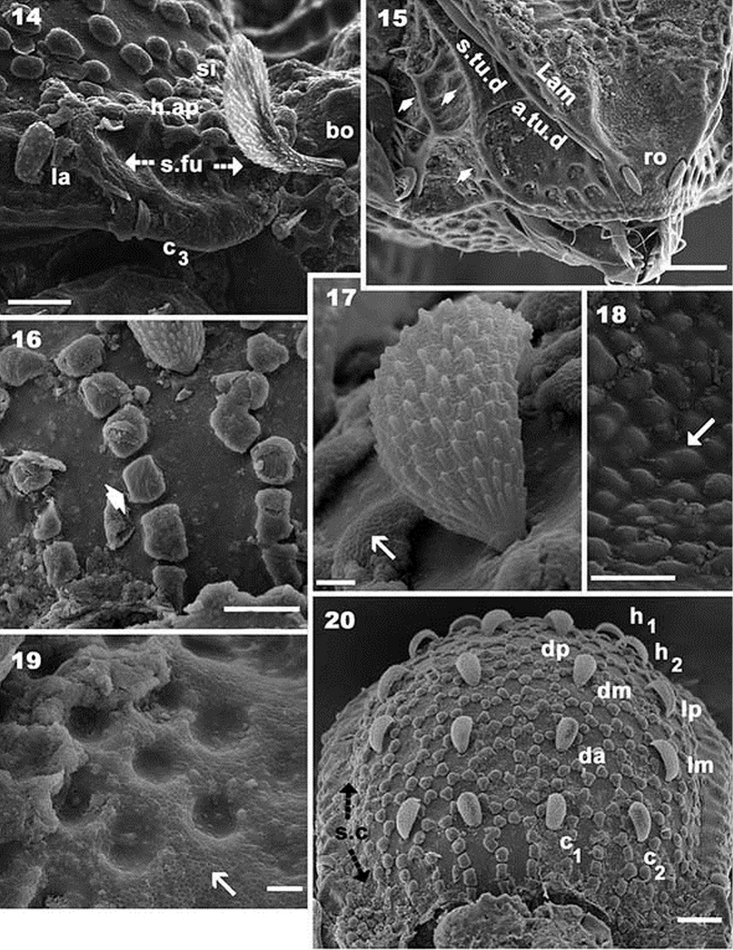
*Yoshiobodes camerunensis* sp. n. Adult, with cerotegumental layer, SEM. **14** lateral view, zone of humeral apophysis **15** anterior prodorsum region **16** cerotegument and cuticular zone: anterior notogaster **17** notogastral setae, frontal view **18** cuticular microsculpture **19** cuticular depressions **20** frontal view, notogaster. Scale bars: 10 μm (**14**); 20 μm (**15**); 10 μm (**16**); 10 μm (**17**); 2 μm **(18)**; 2 μm (**19**); 20 μm (**20**).

**Figures 21–27.**
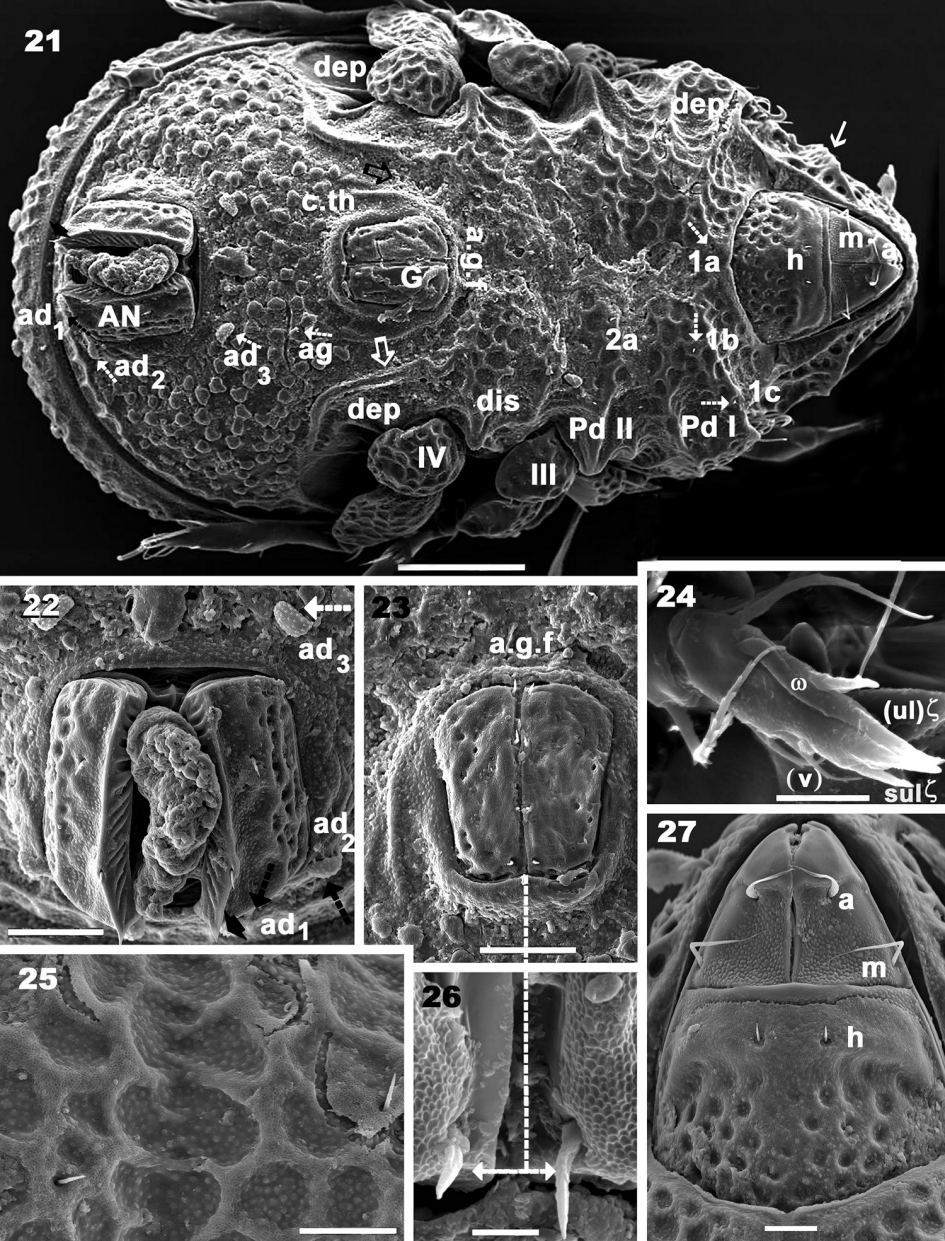
*Yoshiobodes camerunensis* sp. n. Adult, with cerotegumental layer, SEM. **21**ventral view **22** anal plate, ventral view **23** genital plate, ventral view **24** palp **25** epimeral microsculpture with epimeral setae **26** anal setae **27** subcapitulum. Scale bars: 100 μm (**21**); 20 μm (**22, 23**); 10 μm (**26**); 5 μm (**24**); 2 μm (**25**); 10 μm (**27**).

**Figures 28–35.**
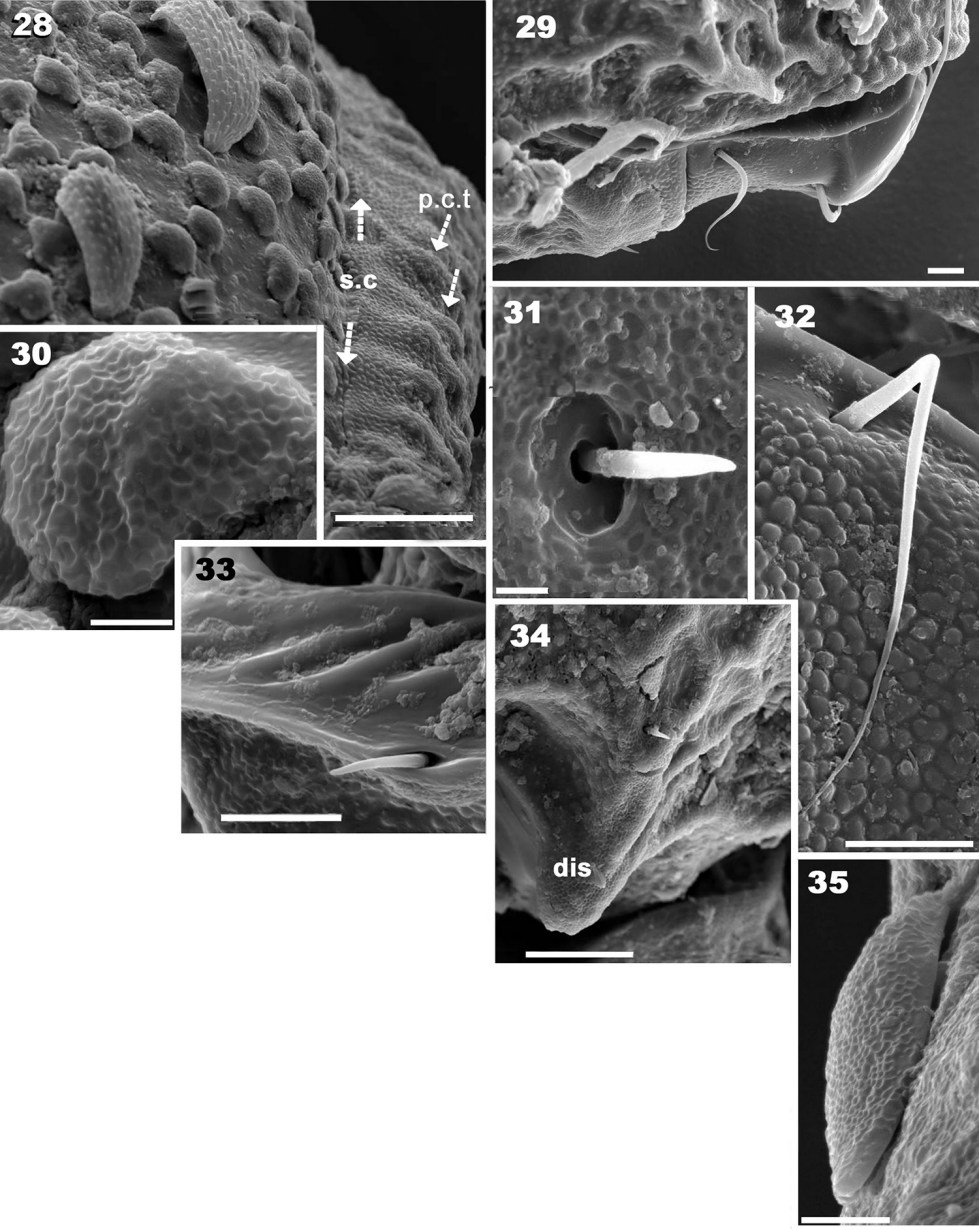
*Yoshiobodes camerunensis* sp. n. Adult with cerotegumental layer, SEM. **28** notogaster, posterolateral **29** subcapitulum, lateral **30** cuticular micrsoculpture covered by cerotegumental layer, dorsal zone **31** subcapitular *h* setae **32** subcapitular setae *m* **33** anal setae **34** discidium and epimeral setae **35** rostral setae, lateral view. Scale bars 20 μm (**28**); 10 μm (**29, 34**); 5μm (**32, 33**); 2 μm (**30, 31**); 1.5 μm (**35**).

*Cuticular microsculpture*. Prodorsum. Posterior zone of *e.i.p* and *p.p.d* round to ovoid depressions (Figure 2). Polyhedral depressions (Figs 6, 15) on anterior zone *e.i.p* near in setae, extending to near ro setae. Remainder of prodorsum with small protuberances (Figures 12, 18). Bothridial zone with large protuberances (Figure 9). Ovoid to irregular depressions of varying size (Figures 6, 15) on lateral zone *Tu*, *s.tu.d* and *Pd I*. Notogaster. Aligned, rectangular to polyhedral protuberances (Figures 6, 16, 20): anterior zone between *c_1_*, *c_2_* setae and *d.sj*; behind *c_1_*, *c_2_* setae and laterally towards *s.c*, ovoid protuberances forming a polyhedral network with 5–7 protuberances (Figures 2, 6, 14, 20, 28). Aligned ridges with small protruberances (Figures 2, 28) in zone between *s.c* and *b.ng.* Ventral zone. Subcapitular zone between *a* and *h* setae with small protuberances similar to Figure 18 (Figures 21, 27, 32). Round depressions (Figure 19). on posterior zone of subcapitulum (Figure 27). Irregular depressions (Figure 25) on epimeral zone (Figure 27). Posterior to genital opening and aggenital, anal and adanal zones, ovoid protuberances forming a polyhedral network (Figure 21). Legs. Large ovoid to round depressions present on basal zone of all femora.

*Setation*. Seta *in* lanceolate, barbate, slightly curving (Figure 8), length 26 μm (22–31); *ro* setae cochleariform, smooth (Figures 12, 15, 35), 10 μm (8–13); *le* setae slightly lanceolate, barbate, covered by cerotegumental layer (Figures 10, 11, 13), 16 μm (11–21). Notogastral setae *c_1_, c_2_, da, dm, dp, lm, lp*, lanceolate-cochleariform, curved, more or less same length (Figures 1, 2, 17, 20, 28), 15 μm (17–22); *c_3_* setae lanceolate, round end with longitudinal shallow grooves (Figures 1, 7), 5.30 μm (4–7); *la, h_3_, p_1_, p_2_, p_3_, h_1_, h_2_* lanceolate, round end with longitudinal shallow grooves (Figure 1), 11 μm (10–13). Subcapitular setae (Figures 21, 27, 31, 32) *a* sigmoid, 9.5 μm (11–8); *m* inclined L-shaped, 20 μm (23–17); spiniform: *h* 2.5 μm (1.8–3.5); epimeral setae (Figures 25, 34), 0.7 μm (0.4–1.7); *ge* (Figures 21, 23, 26), 7 μm (10–6); *ag* (Figure 21), 7 μm (5–10); *an* (Figure 22), 7 μm (12–4). Adanal setae *curved lanceolate-cochleariform* (Figure 21) *ad_1_, ad_2_, ad_3_* 15 μm (17–13).

*Prodorsum.* Very complex, described from different angles in order to properly interpret the structure. Lateral view (Figure 1) and slightly posterolateral inclination (Figure 2): elevated interlamellar process (*e.i.p*) at the same level as elevated zone of notogaster; forward directing *in* setae situated in a depressed zone (Figure 8); posterior prodorsal depression (*p.p.d*) clearly visible in inclined lateral view (Figure 2). Cuticular microsculpture and shallow lamellar furrow (*l.l.f*) well visible (Figure 2). Lamellar zone (Figures 10, 11, 13): positioning of *le* setae and their particular shape clearly observed; lamellae in anterior zone lacking lamellar tips, anterior zone is connected by a bridge (Figure 11 indicated by arrow). Anterior lateral view (Figure 15): cuticular microsculpture of *s.tu.d* zone and between *Pd I* and *Tu* is clearly visible; the zone between seta *ro* is more or less smooth. Bothridia: cup-shaped, with smooth ovoid bothridial ring, incomplete, with bothridial tooth (Figures 2, 14). Sensillus (*si*) fan-shaped, barbed, directing upwards (Figures 1, 2, 6, 9, 14).

Frontal view (Figures 6, 12): triangular, with depressed central zone (Figure 6, indicated by thick arrow). Both sides of *e.i.p* rounded, with depressed zone where *in* setae are located. Cuticular microsculpture easily observed on depressed central zone of *e.i.p*; *l.l.f* clearly visible; *le* setae hardly discernible; *ro* setae well visible; rostral margin smooth. *Tu* (Figure 6) expanded laterally towards the prodorsal margin, extending to the level of *Pd I*.

*Notogaster.* Shape: oval in dorsal view; *d.sj* narrow, well-delimited, curving slightly backwards (Figures 2, 3); anterior lateral zone: humeral apophysis (*h.ap*) extending forward, overlapping posterior bothridial zone (Figures 2, 3, 5 14); notogastral anterior depression (*n.a.d*) absent. Frontal view: convex in central zone up to cicumgastric furrow (*s.c*); flat from *s.c* to *b.ng*, slightly tilted downward (Figures 2, 6, 20); *s.c* clearly delimited (Figures 6, 20), running laterally between *c_2_, lm, lp, h_2_, h_1_, dp* and *h_3_, p_3_, p_2_, p_1_*. Setae *c_3_* and *la* present on *h.ap*, with conspicuous depressed zone (*s.fu*) lodging the sensillus after leg folding (protection mechanism) (Figure 14) (Fernandez et al. 2013); *c_3_* setae placed beneath the *s.fu*, while *la* are placed behind. Cuticular microsculpture: rectangular to polyhedral protuberances are clearly delimited in the anterior zone between *d.sj* and setae *c_1_, c_2_* and inwards to *s.c* (Figures 16, 20). Setae *c_1_, da*, and *dm* are more or less aligned; while *c_2_, lm, lp, h_2_*, and *h_1_*, are arranged in an arc (Figures 6, 20).

Lateral view: convex (Figure 1, 5), setae *la, h_3_, p_3_, p_2_, p_1_* situated between *s.c* and *b.ng*; setae *c_3_* are situated further down from this setal alignment (Figures 1, 14). Fifteen pairs of setae: *c_1_, c_2_, c_3_, da, dm, dp, la, lm, lp, h_1_, h_2_, h_3_, p_1_, p_2_, p_3_*; only lyrifissures *im* and *gla* clearly visible between *lm* and *lp* setae (Figure 5). Clearly visible *s.c* in lateral posterior zone (Figure 28); cuticular microsculpture below *s.c* different to zone above, and parallel cuticular thickening (*p.c.t*) situated between *s.c* and *bng*, clearly discernible (Figure 28).

*Lateral region* (Figures 1, 5, 14). A thorough study of the lateral aspect was imperative for observation and interpretation of several structures. Conical *e.i.p* inclining slightly upwards (Figures 1, 5); *lam* clearly discernible (Figures 1, 5, 10, 11, 13, 15); *le* inserted on *lam*, behind level of *ro* setae (Figure 15); no lamelar tips present; *le* setae inserted some distance from where the apical part of *lam* reaches the rest of prodorsum; this zone forming a bridge where *le* setae can be concealed (Figures 10, 15); large, laterally expanded *Tu* at same level as *Pd I* (Figure 15); *Tu* with upward curving margin; several depressions (Figure 15) visible on *Tu* and zone between *Tu* and *Pd I*, with variation in shape and depth (Figure 15 indicated by arrows); *s.tu.d* deeply concave; anterior tutorial depression (*a.tu.d*) (Figure 5) and other small depressions present (Figure 15); *Pd I*: large extended lamina, rounded apex; immediately behind *Pd I* apex, conspicuous round to polyhedral cuticular depression (Figure 1 indicated by dashed arrow);in posterior zone of *Pd I*, near *Pd II*, short deep longitudinal grooves separated from each other by longitudinal depressions (Figure 1, indicated by arrow). *Pd II*: small lamina, rounded apex; *dis* a triangular protuberance (Figure 34). Many circular to ovoid depressions (*dep*), delimited by cuticular thickenings, occurring behind, on top of and on lower part coxa IV up to genital opening (Figures 1, 5, 21).

*Ventral region.* Cuticular microsculpture obviously different on epimeral, aggenital, and adanal zones (See Cuticular microsculpture). Subcapitular setae *a, m, h* (Figures 21, 27, 31, 32, 34) differing in shape and length (see Setation); setae *h* similar to epimeral, genital, aggenital setae (Figures 22, 23, 25, 33), all spiniform; epimeral setae shorter than others, difficult to observe.

*Epimeres* well defined by furrows, easily discernible both in animals with cerotegumental layer (Figure 21) and without (Figure 4). Epimeral borders clearly visible (Figure 4); epimeral chaetotaxy 3-1-3-3, but variations exist due to some setae not being clearly visible, in asymmetric position, or lost; apodemes 1, 2, sj and 3 clearly visible (Figure 4); epimera 1, 2; 3 and 4 fused. Genital opening on elevated zone (Figure 21); surrounded anteriorly by a semicircular cuticular thickening (*c.th*) (Figure 21) extending to posterior zone, but not completely surrounding genital opening; depressed zone between cuticular thickening and elevated zone of genital opening; deep anterior furrow (*a.g.f)* (Figures 4, 21) in front of genital opening, this depressed zone extends to the outside of *c.th*. Four pairs of genital setae in single line (Figures 21, 23). Posterolateral aggenital setae, genital opening far from *ad_3_* setae; very different in shape and size (Figure 21). Three pairs of adanal setae. Anal plate sharply tipped (Figure 22); lyrifissure *iad* situated laterally, hardly discernible (Figure 4). Many circular to ovoid depressions (*dep*), behind coxa IV.

*Legs*. Setal formulae (trochanter to tarsus) (Table 1) Legs. I: 1-4-3(1)-4(2)-15(2); II: 1-4-3(1)-3(1)-15(2); III: 2-3-1(1)-2(1)-15; IV: 1-2-2-2(1)-12 (trochanter to tarsus).

**Table 1.** Leg setae and solenidia of *Yoshiobodes camerunensis* sp. n.

Leg	Femur	Genu	Tibia	Tarsus
*I*	*(l). d, dv*	*(l),v*’	*(l),(l)*	*(pv),s,(a),(u),(p), (tc), (ft)*,ε,*it*”
		*σ*	φ*_1,_* φ*_2_*	ω*_1,_* ω*_2_*
*II*	*(l), d, dv*	*(l),d*	*(l), v*’	*(pv), s, (a), (tc), (u), (p), (ft),(it)*
		*σ*	φ	ω ω
*III*	*l,v*	*d,v,l*’	*l*’	*(pv), s, (a), (tc), (u), (p), (ft),(it)*
		*σ*	φ	
*IV*	*d,ev*	*d, l*’	*(v)*	*(pv),(u),(p),ft”,s,(a), (tc)*
			*σ*	

#### Remarks.

The positioning of the *le* setae during activation of the protection mechanism is interesting: these setae are shielded under the lamellae, but are also further protected by the cerotegumental layer (Fig. 10). Protected by the external margin of *Lam* (figure 1), and concealed in the deepest zone of the *s.tu.d*, Legs I are difficult to study. *Yoshiobodes camerunensis* is the first species of this genus from the Afrotropical region. *Y. irmayi* (Balogh & Mahunka, 1969), redescribed by Reeves 1997, is close to *Yoshiobodes camerunensis* sp. n. Principal similarities: presence of *p.p.d* on prodorsum; rectilinear microsculpture between *d.sj*; microsculpture *c_1_*, *c_2_* setae and behind setae *c_1_, c_2_*; number of notogastral setae; shape of notogastral setae; shape of *in* setae. Principal differences: prodorsal cuticular microsculture, shape of prodorsum; characteristics of *l.l.f*; shape and characteristics of *ro* setae; shape and characteristics of *le* setae; microsculpture of epimeral zone; structure *s.fu*.

### Rugocepheus costaricensis sp. n.

Taxon classificationAnimaliaSarcoptiformesCarabodidae

http://zoobank.org/97AA08B8-332F-4C20-8631-E7EDE2CEC4E6

Figures 36–39 , 40–42 , 43–52 , 53–61 , Table 2

#### Etymology.

The specific epithet is derived from Costa Rica, country of origin of the type material.

#### Material examined.

**Holotype**. Adult female “CCR 0978 Tu 11 Costa Rica Turrialba foret naturelle du catie alt. 560 m. Triage d’humus coté est surface nid d’Atta au pied de *Castilla elastica* 1.IX. 1978. Leg. P.WERNER” Deposited in the Ccllection of the MHNG, Switzerland, preserved in 70% ethanol. **Paratypes.** 2 adult females, same locality and date of holotype, deposited in the Ccllection of the MHNG, Switzerland, preserved in 70% ethanol.

**Figures 36–39.**
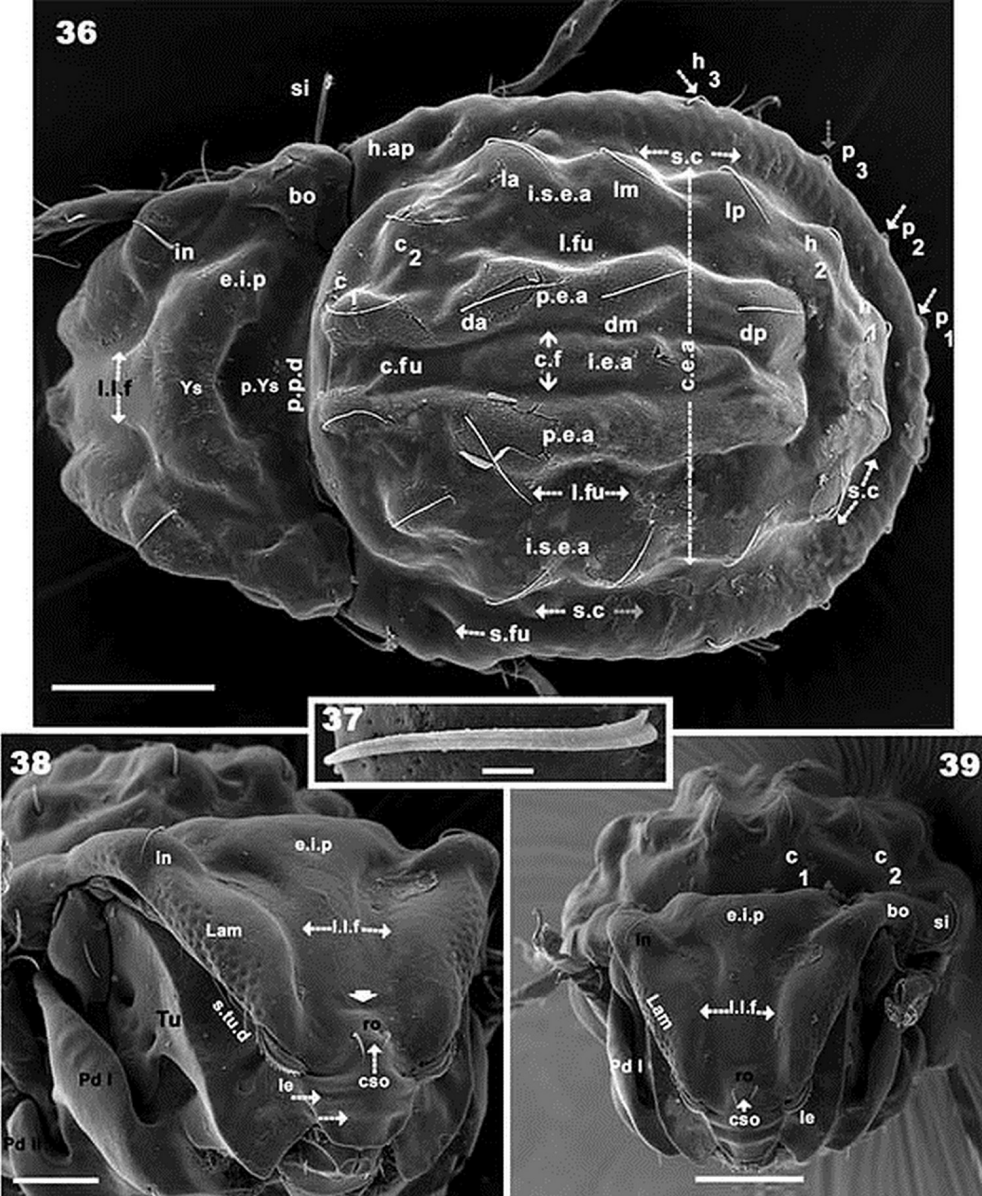
*Rugocepheus costaricensis* sp. n. Adult (with cerotegument), SEM. **36** dorsal view **37** *in* setae **38** fontal inclined view **39** frontal view. Scale bars: 100 μm (**36**); 5 μm (**37**); 50 μm (**38**); 100 μm (**39**).

#### Diagnosis (adult female).

Body and legs entirely covered by simple porous cerotegumental layer. Integumental microsculpture over entire body: irregular, small tuberculate. *Prodorsum*. More or less triangular in dorsal view; deep low lamellar furrow delineating Y-shaped structure; elevated interlamellar process with superior flat zone; *CSO* present; rostrum beak-shaped; tutorium and Pedotecta I expanded laterally; small triangular discidium; supratutorial depression deep, with several rounded depressions. Lamellae without lamellar tip, forming bridge concealing *le* setae. Smooth ring-shaped bothridium; bothridial tooth present; sensillus barbate. Fourteen pairs of notogastral setae *c_1_, c_2_, da, dm, dp, la, lm, lp, h_1_, h_2_, h_3_, p_1_, p_2_, p_3_*. Four notogastral furrows present: paired central longitudinal furrow; one lateral unpaired semicircular furrow; another unpaired semicircular furrow delimiting an unpaired elevated central area (devoid of setae); a pair of elevated areas (with *c_1_, da, dm, dp*); unpaired semicircular elevated area (with *c_2_, la, lm, lp, h_1_, h_2_*). Anterior genital furrow clearly observed; epimere 4 borders elevated; genital plate situated in elevated zone surrounded by furrow. Epimeral chaetotaxy 3-1-3-3; long epimeral setae. Four pairs of genital setae in a single line; crescent-shaped structure anterior to anal plate; anal plate with small sharp tip; aggenital and adanal setae more or less similar in length. Subcapitulum diarthric, three pairs of highly different setae *a, m, h*. Mentum complex.

#### Description.

*Measurements*. SEM: females 501 μm (489–515) × 270 μm (267–286). Light microscopy: females 506 μm (490–518) ×282 μm (276–301).

*Colour*. Specimens without cerotegument: females light brown to brown.

*Cerotegument*. Simple layer (±0.7 μm) (Figures 47, 49, 50); uniformly covering entire body and legs. Slightly irregular surface (Figure 50). Large number of pores observable on the surface, porous (0.4–0.7 μm) diameter (Figures 44, 46, 47, 50).

*Integument.* Microsculpture simple, covering entire body: irregular, small tuberculate (Figure 49); tubercules (1–2.5 μm). Only lateral anterior lamellar zone presenting different microsculpture: round to ovoid depressions (Figures 38, 39, 43).

*Setation.* Setae in lanceolate (resembling leaf of *Salix* spp.), length 30 μm (28–34) (Figure 37); *ro* setae lanceolate, 13 μm (11–14) (Figure 44). Setae *le* lanceolate, slightly curved, basally and medially serrate, 23 μm (21–25) (Figure 45). Notogastral setae; *c_1_, c_2_, da, dm, dp, la, lm, lp ,h_1_,h_2_* aciculiform, 51 μm (41–61) (Figure 46); *h_3_, p_1_, p_2_, p_3_*, 25 μm (23–27) (Figures 36, 46). Simple: ag, 20 μm (17–22) (Figure 61); ad 20 μm (17–22) (Figure 61); ge 17 μm (15–19) (Figure 60); epimeral 18 μm (15–21) (Figure 53). Spiniform: *an* 10 μm (11–8) (Figure 55); *m* 3.5 μm (3–4) (Figure 57); Setae *a* setiform, 7 μm (5–9) (Figure 56); *h* setae L-shaped, barbate, 19 μm (18–21) (Figure 58).

**Figures 40–42.**
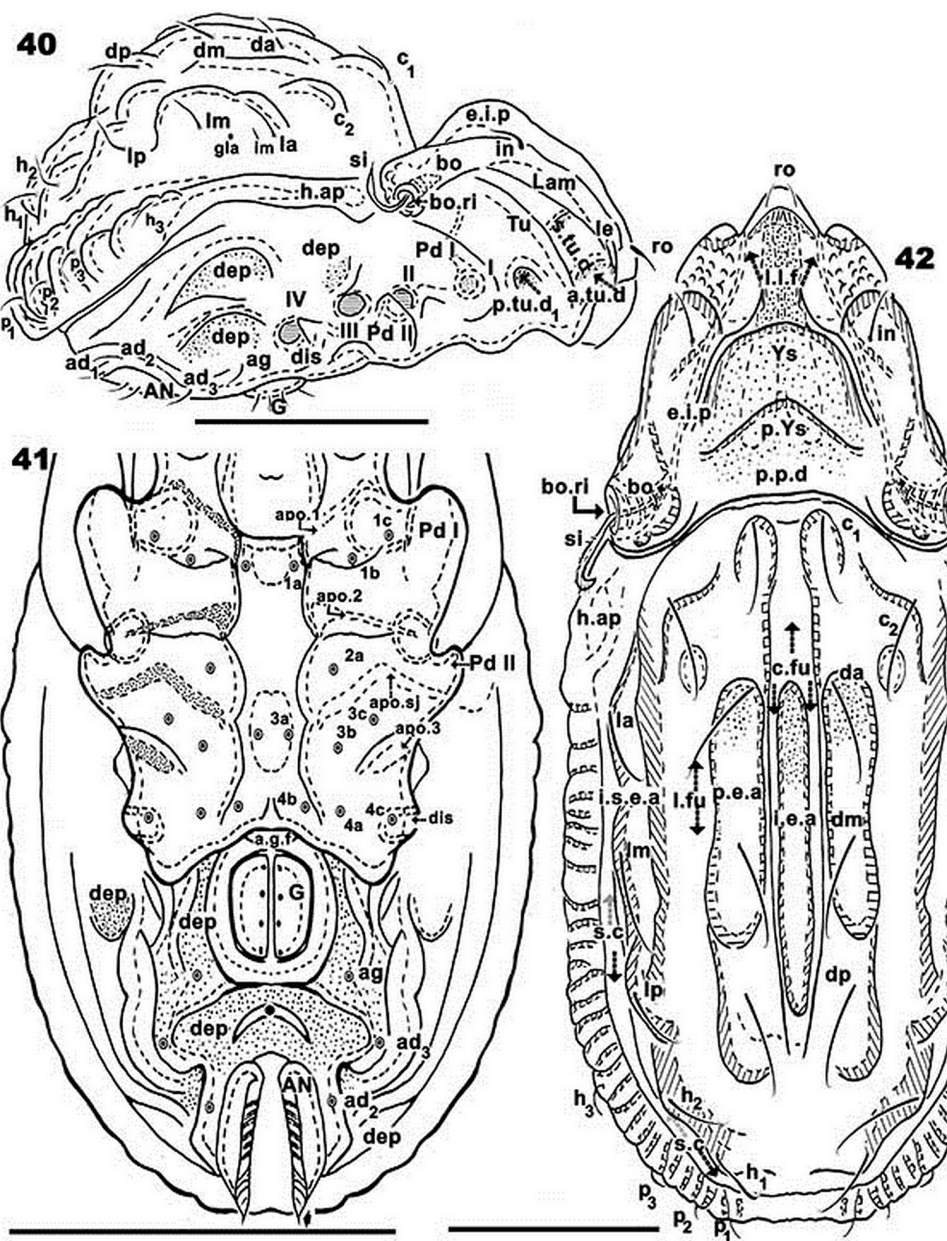
*Rugocepheus costaricensis* sp. n. Adult (with cerotegument) optical microscopy. **40** dorsal view **41** ventral view **42** lateral view.

*Prodorsum*. Very complex. For proper understanding of structures, descriptions from various angles/views are included. Dorsal view (Figure 36). More or less triangular with lateral polyhedral expansion at level of *bo* and *in* setal level insertion; anterior expansion of *Tu* (Figure 36) clearly visible. Deep *l.l.f* delineates Y-shaped structure (*Ys*); posterior of *Ys* with depressed rounded zone (*p.Ys*) extending laterally in *p.p.d* *; *ro* setae and *CSO* clearly visible. Beak-shaped rostrum; sensillus barbate.

Frontal view (Figure 39). More or less triangular; *e.i.p* elevated with flat superior medial zone (Figure 38); conspicuous *l.l.f* running to posterior zone of *e.i.p*; from *ro* setal zone, *l.l.f* delineates a Y-shaped structure. On posterior of *e.i.p* the *l.l.f* delimiting a large ear shaped structure where *in* setae are situated. Lamellae (*lam*), running laterally, internal margin delimited by *l.l.f*; *le* setae on the anterior zone of *lam*; *le* setae inserted behind *ro* setal insertion level; small transversal depression posterior to *ro* setae (Figure 38 indicated by thick arrow); *CSO* present anterior to *ro* setal insertion. Rostral zone extended to rounded beak-shape with several transversal semicircular furrows (Figure 38 indicated by dashed arrow).

**Figures 43–52.**
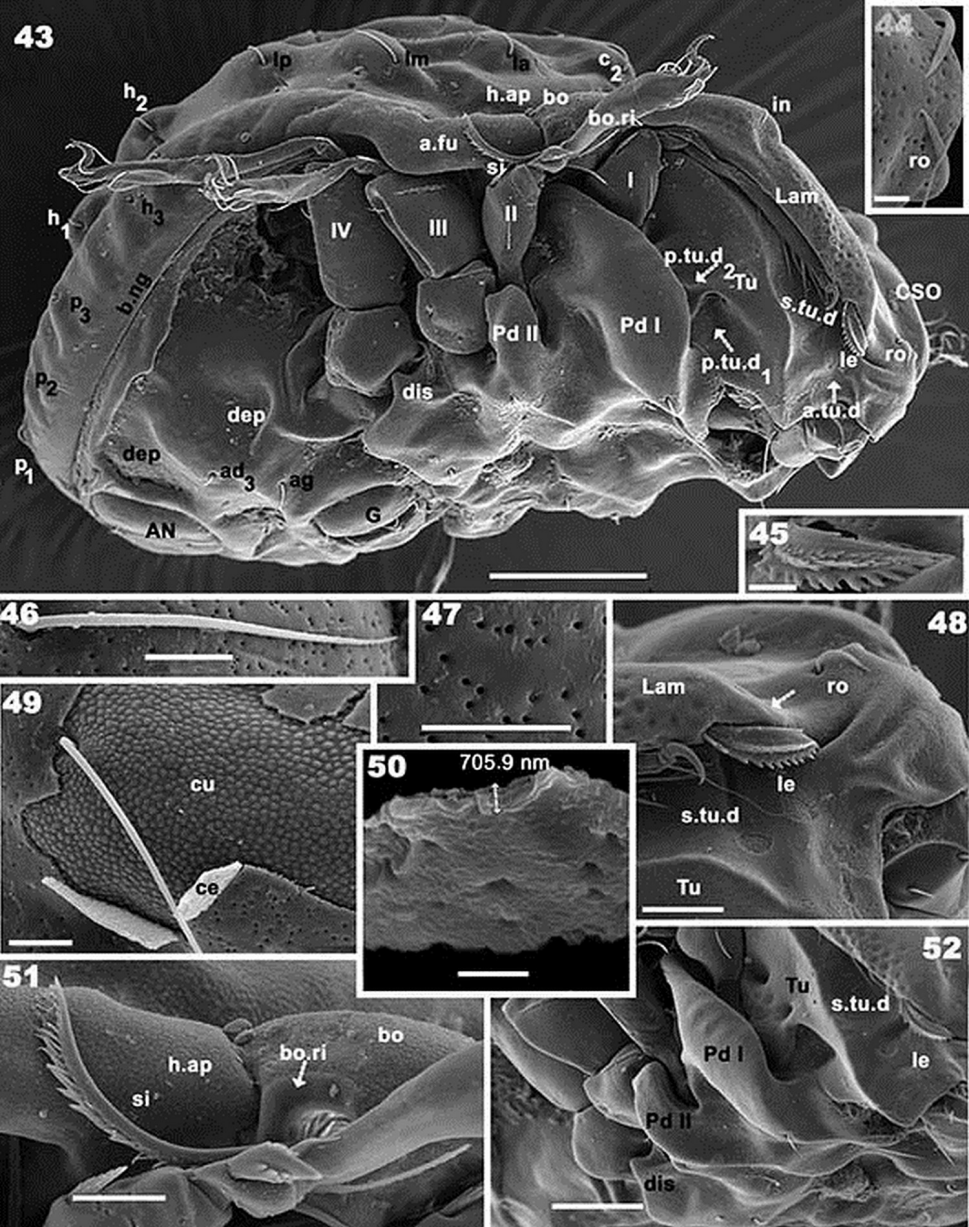
*Rugocepheus costaricensis* sp. n. Adult, (with cerotegument) SEM. **43** lateral view **44** rostral zone **45** *le* setae; **46** notogastral setae **47** notogastral cerotegumental layer, ventral view **48** anterior prodorsal zone **49** notogastral cerotegumental layer, ventrolateral view **50** cerotegumental layer **51** bothridial zone **52** ventrolateral inclined zone. Scale bars: 100 μm (**43**); 5 μm (**44**); 10 μm (**45**); 5 μm (**46**); 10 μm (**47**); 20 μm (**48**);10 μm (**49**); 2 μm (**50**); 20 μm (**51**); 50 μm (**52**).

Lateral inclined view (Figures 38, 43). Elevated *e.i.p* with flat superior zone; *lam* clearly delimited by conspicuous *l.l.f*; particular cuticular microsculpture of round to ovoid depressions externally to *lam.* Elevated ear-shaped structure where *in* setae are situated; *ro* setae, *CSO*, and beak-shaped rostral zone, easily observed. *Tu* expanded laterally and anteriorly; *Pd I*: large expanded ovoid structure; several depressions (*p.tu.d, p.tu.d_1_*) between *Tu* and *Pd I*; *s.tu.d* a conspicuous depression, running parallel between *lam* and *Tu*, with internal round depression (*a.tu.d*); *le* setae inserted on anterior zone of *lam*; *lam* zone anterior to *le* insertion, lacking lamellar tip, forming a bridge concealing *le* setae. Bothridium cup-shaped, smooth bothridial ring, incomplete, with bothridial tooth.

*Notogaster* (Figure 36). Oval, with fourteen pairs of setae: *c_1_, c_2_, da, dm, dp, la, lm, lp, h_1_, h_2_, h_3_, p_1_, p_2_, p_3_*. Four furrows present: paired central longitudinal (*c.fu*) furrows; one lateral unpaired semicircular furrow (*l.fu*), and one unpaired semicircular (*s.c*) furrow; an unpaired elevated central area (*i.e.a*) is defined by paired *c.fu*. A pair of elevated areas (*p.e.a*) defined by *c.fu* and *l.fu*; an unpaired semicircular elevated area (*i.s.e.a*) defined by *l.fu* and *s.c*. The *i.e.a* is devoid of setae; *p.e.a* with *c_1_, da, dm, dp*; *i.s.e.a* with *c_2_, la, lm lp, h_1_, h_2_*. Setae *h_3_, p_1_, p_2_, p_3_* situated between *s.c* and *b.ng.* Setae *c_1_, c_2_, da, dm, dp, la, lm, dp, h_1_, h_2_* situated on dorsal protuberances (*d.pr*), while h_3_, p_3_, p_2_, p_1_ are inserted on lateral thickenings (Figure 43); lyrifissure *im* and *gla* clearly visible (Figure 40).

*Lateral region* (Figures 43, 48). *Lam* (Figure 48) with elevated zone bearing *in* setae; towards anterior of *le* setae, lacking lamellar tip, forming a bridge, permitting concealment of setae; *s.tu.d* a deep depression; *tu* clearly delimited by prominent thickening; *a.tu.d*.,*p.tu.d_1_*, and *p.tu.d_2_* between *tu* and *Pd I*. Rostrum beak-like. Inferior curved margin of lamella continuous with inferior bothridial part; both structures related to *s.tu.d*, permitting concealment of tarsus, tibia and dorsal area of genu and femur of leg I during leg-folding (protection mechanism). *Pd I*: large curved extended lamina. *Pd II*: small rectangular to polyhedral lamina. Humeral apophysis (*h.ap*): large polyhedral structure, conspicuous oblique posterior furrow on surface (*s.fu*); anterior *h.ap*. zone overlapping posterior part of bothridial zone. Discidium (*dis*): small triangular structure. Several large ovoid depressions behind acetabulum IV and posterolateral to genital and anal openings.

**Figures 53–61.**
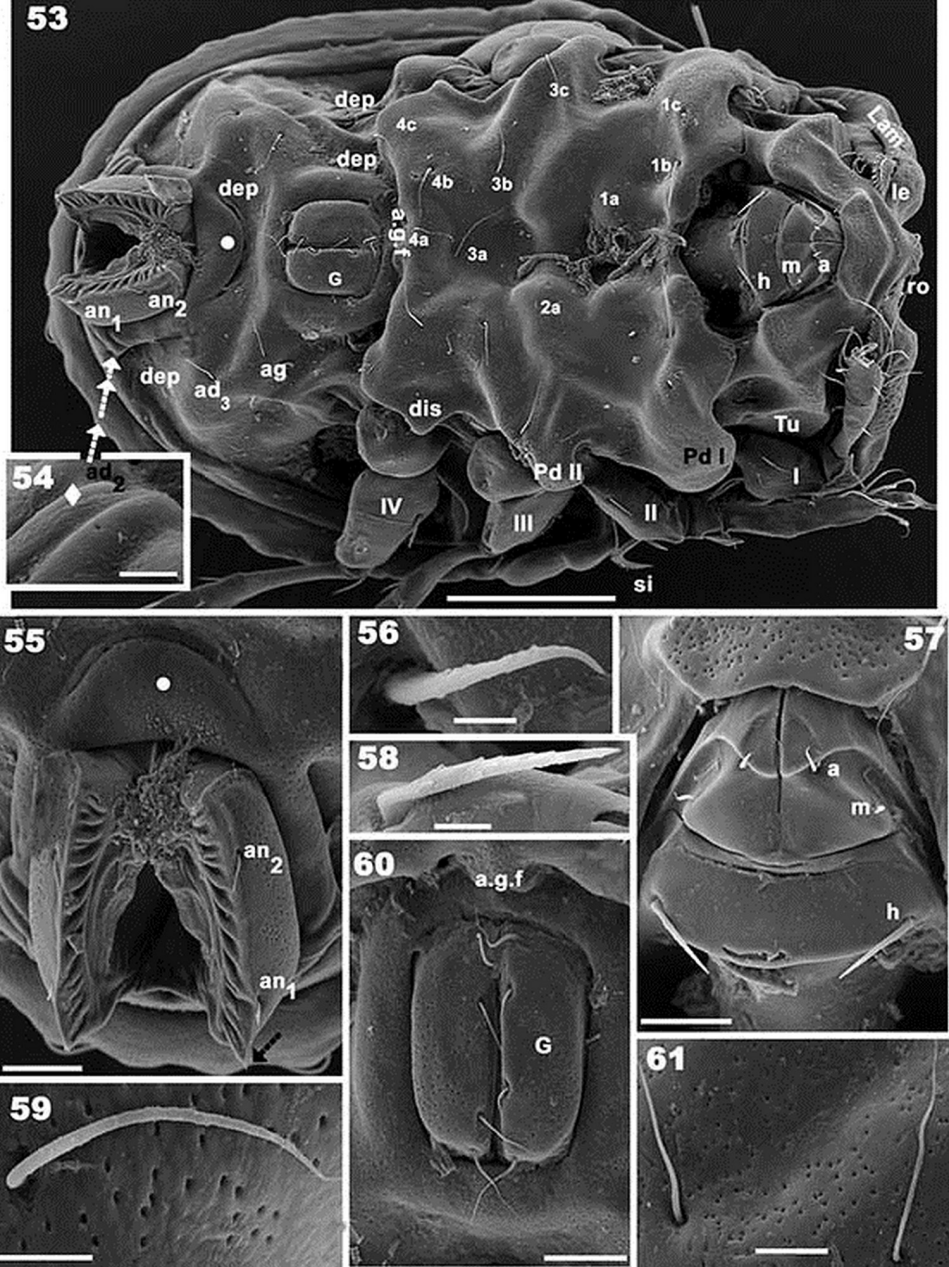
*Rugocepheus costaricensis* sp. n. Adult, (with cerotegument) SEM. **53** ventral view **54** *ad_2_* setal zone **55** anal zone **56** *a* subcapitular setae **58** *h* subcapitular setae **57** subcapitulum **59** epimeral *3b* setae **60** genital zone **61** aggenital, adanal setae. Scale bars: 100 μm **(53)**; 5 μm (**54**); 20 μm (**55**); 2 μm (**56**); 5 μm **(57)**; 5 μm (**58**); 5μm (**59**); 20 μm (**60**); 10 μm (**61**).

*Ventral region*. Epimeral zone more or less smooth with large elevations and depressions. Paraxial zone of epimera 1 and 2 with longitudinal furrow; large paraxial depression behind *bo.sj* . Epimere 4 posterior border elevated. Anterior genital furrow (*a.g.f*) well visible (Figure 53); genital plate situated on elevated zone surrounded by furrow (Figure 60). Epimeral chaetotaxy 3-1-3-3 (Figure 53); long epimeral setae (Figure 59). Four pairs of genital setae in a single line (Figure 60). Crescent-shaped structure anterior to anal plate (Figure 55 indicated by large dot); anal plate with small sharp tip. Aggenital and adanal setae more or less similar in length (Figures 54, 61). Subcapitulum diarthric (Figure 57); three pairs of highly differing setae *a*, *m*, *h* (Figure 57). Mentum complex.

*Legs* (Table 2). I(1-3-3-4-16-1) (1-2-2); II(1-4-2-3-16-1) (1-1-2); III(2-3-1-2-14-1) (1-1-0); IV(1-2-2-2-12-1) (legs similar to other species, therefore not illustrated).

**Table 2.** Leg setae and solenidia of *Rugocepheus costaricensis* sp. n.

Legs	Femur	Genu	Tibia	Tarse	Claw
I	*v (l)*;	*(v) d*	*(l),v*	*(pv); s; (a); (u); (p); (it); (tc); (ft)*; ε	1
		*σ*	φ*_1,_* φ*_2_*	ω*_1,_* ω*_2_*	
II	*(l) d*	*v l*	*v (v)*;	*d Ad; (pv); s; (a); (u); (p); (it); (tc); (ft).*	1
		*σ*	φ	ω*_1,_* ω*_2_*	
III	*l*;	*d*	*v (l)*	*(v) ft; (tc); (it); (p); (u); (a); s; (pv).*	1
		*σ*	φ		
IV	*d*	*v d*	*(l).*	*v ft; (tc); (p); (u); (a); s; (pv*	1
			φ		

#### Remarks.

*Rugocepheus costaricensis* sp. n. displays important differences to *Rugocepheus joffrevillei* Fernandez, Theron & Rollard, 2013 and *R. formosus* Mahunka, 2009. Principal differences: beak-shaped rostrum; distribution of furrows and elevated areas on dorsal zone of notogaster, central elevated area without setae; ventral zone with discidium differing in shape; genital and anal zone very different.

#### Discussion.

Using SEM allows significant progress in detailed descriptions, as the small body size, morphological characteristics, and complex topology makes *Yoshiobodes* a difficult genus to study. This complexity is compounded by brief, somewhat cryptic original descriptions and illustrations. Reeves (1997), contributed much to our understanding of this genus, specifically due to studies of both adults and immatures. Reeves also originally pointed out the following characters with reference to the adult prodorsum of *Yoshiobodes*: “Dorsosejugal depression deep, slit-like, widest medially” (page 316) (in our series of papers on the revision of the family Carabodidae, this depression is designated as the “posterior prodorsal depression (*p.p.d*)” Fernandez et al. 2013), but this structure was not noted again until this present paper. The analysis by Reeves of the work done by Bellido (1978) is noteworthy as he analyses the depression observed on the prodorsum in protonymphs, deutonymphs and tritonymphs of *Carabodes*. Reeves (1997) indicates: “The scalloped edged depression on the prodorsum of protonymphs, deutonymphs and tritonymphs appears similar to the foveate sclerite found in immatures described by Bellido (1978) of *Carabodes willmanni* Bernini, 1975.”

The most recent generic diagnosis by Ermilov et al. 2014 is based on data from Mahunka (1986) and additions by authors, but the type specimen, *Y. irmayi* (Balogh & Mahunka, 1969) does not seem to have been studied. SEM and optical microscopy studies by Reeves (1997) on adults as well as ontogenetic studies, were also not discussed. Reeves 1997 indicated that, on comparison, “a specimen of *Y. irmayi* from St. Lucia (on loan from the Hungarian Natural History Museum) to North American material showed them to be conspecific”.

For the purpose of this present paper, *Yoshioiodes* is considered only on the basis of Balogh and Mahunka (1969) (*Carabodes irmayi*) and Reeves (1997). We await further studies on type specimens of the following subgenera: Yoshiobodes (Yoshiobodes) Mahunka, 1986, type species *Carabodes irmayi* Balogh & Mahunka, 1969; Yoshiobodes (Berndobodes) Mahunka, 1986 type species, *Berndobodes spiculifer* Mahunka, 1986; Yoshiobodes (Dongnaiobodes) subgen. n. type species *Yoshiobodes hexasetosus* Ermilov, Shtanchaeva, Subías & Anichkin, 2014. As part of the ongoing revision of the Family Carabodidae (started in 2013), we have studied the type material of *Berndobodes spiculifer* Mahunka, 1986, and further information on this genus will be included in an upcoming revisionary paper.

## Supplementary Material

XML Treatment for Yoshiobodes camerunensis

XML Treatment for Rugocepheus costaricensis
